# Multiple Functions of the New Cytokine-Based Antimicrobial Peptide Thymic Stromal Lymphopoietin (TSLP)

**DOI:** 10.3390/ph9030041

**Published:** 2016-07-05

**Authors:** Louise Bjerkan, Andreas Sonesson, Karl Schenck

**Affiliations:** 1 Department of Oral Biology, Dental Faculty, University of Oslo, PB 1052 Blindern, N-0316 Oslo, Norway; louise.bjerkan@medisin.uio.no; 2 Division of Dermatology and Venereology, Department of Clinical Sciences Lund, Lund University, BMC, Tornavägen 10, SE-22184 Lund, Sweden; andreas.sonesson@med.lu.se; 3 Dermatology and Venereology, Skane University Hospital, Lasarettsgatan 15, SE-22185 Lund, Sweden

**Keywords:** TSLP, AMP, immunoregulation

## Abstract

Thymic stromal lymphopoietin (TSLP) is a pleiotropic cytokine, hitherto mostly known to be involved in inflammatory responses and immunoregulation. The human *tslp* gene gives rise to two transcription and translation variants: a long form (lfTSLP) that is induced by inflammation, and a short, constitutively-expressed form (sfTSLP), that appears to be downregulated by inflammation. The TSLP forms can be produced by a number of cell types, including epithelial and dendritic cells (DCs). lfTSLP can activate mast cells, DCs, and T cells through binding to the lfTSLP receptor (TSLPR) and has a pro-inflammatory function. In contrast, sfTSLP inhibits cytokine secretion of DCs, but the receptor mediating this effect is unknown. Our recent studies have demonstrated that both forms of TSLP display potent antimicrobial activity, exceeding that of many other known antimicrobial peptides (AMPs), with sfTSLP having the strongest effect. The AMP activity is primarily mediated by the C-terminal region of the protein and is localized within a 34-mer peptide (MKK34) that spans the C-terminal α-helical region in TSLP. Fluorescent studies of peptide-treated bacteria, electron microscopy, and liposome leakage models showed that MKK34 exerted membrane-disrupting effects comparable to those of LL-37. Expression of TSLP in skin, oral mucosa, salivary glands, and intestine is part of the defense barrier that aids in the control of both commensal and pathogenic microbes.

## 1. Introduction

Thymic stromal lymphopoietin (TSLP) was first identified in the culture supernatant of a murine thymic stromal cell line and was shown to support B-cell growth and development [1]. The human homologue of TSLP was cloned and characterized in 2001 and showed only 43% amino acid sequence identity with mouse TSLP [2]. Despite the low amino acid sequence homology, human and murine TSLP are functionally similar [3]. Human TSLP was identified as a four-helix bundle cytokine containing six conserved cysteine residues and multiple sites for N-linked glycosylation [2,4].

Two variants of human TSLP peptides are expressed. Most studies hitherto have been focused on a long form of TSLP (lfTSLP), while translation of a short form (sfTSLP) has been reported only recently [5]. lfTSLP is inducible and associated with inflammation, and sfTSLP is constitutively expressed and has an inhibiting effect on dendritic cells (DCs) [5,6]. Lately, we have shown that both TSLP forms also act as strong antimicrobial peptides (AMP) [5,7]. Here, we describe the variants of human TSLP and their expression in the human body, summarize their activity on different elements of the immune system, describe their qualities as an AMP, and outline some of the mechanisms behind their antimicrobial effects.

## 2. TSLP Variants

**Figure 1:**
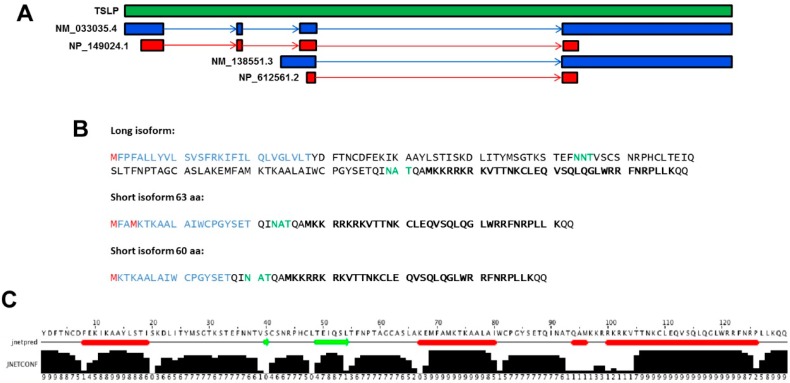

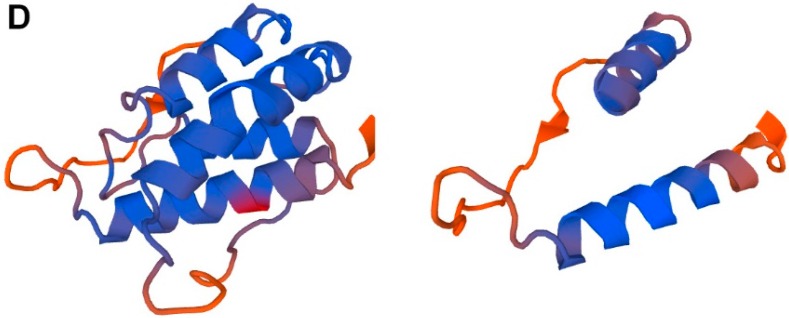
TSLP transcript variants and protein isoforms. (**A**) Graphics showing the TSLP gene (green), the long form, and short form transcripts (blue), and the protein products (red). Long (NM_033035.4, NP_149024.1) and short (NM_138551.3, NP_612561.2) transcript and protein variants, respectively, are indicated (NCBI). (**B**) Amino acid sequence of human TSLP isoforms. The putative signal sequences of the TSLP isoforms are marked in blue, and the mature protein in black. N-linked glycosylation sites are marked green, and methionine start codons are marked red. Bold black characters indicate the position of MKK34. (**C**) JNet secondary structure prediction of lfTSLP based on the amino acid sequence. Helices are marked as red tubes, and sheets are marked as green arrows. JNETCONF: The confidence estimate for the prediction, high values indicate high confidence. Modified from the web-based application Jpred (The Barton Group, School of Life Sciences, University of Dundee, UK). (**D**) Three-dimensional structure (Swiss-model, [9]) of lfTSLP (**left**) and sfTSLP (**right**).

Three transcript variants of human TSLP are annotated in the RefSeq (Reference Sequence) database (National Center for Biotechnology Information, National Library of Medicine, Bethesda, MD, USA), two long and one shorter variant, but only one of the long (variant 1) and the short (variant 2) variants give rise to coding RNA. Variant 1 consists of four exons and variant 2 is two 5′ exons shorter but contains an alternate 5′ exon compared to variant 1 (Figure 1A). The relevance of distinguishing between TSLP variants in mice is currently uncertain as a murine short TSLP variant has not been described or annotated in RefSeq so far.

The long form (lfTSLP) encodes a 159 amino acid (aa) protein. The short form (sfTSLP) encodes a sequence that is identical in the C terminal region of long TSLP and consists of 63 aa (UniProt entry: G3XAM8) and/or a 60 aa (UniProt entry: Q96AU7) (Figure 1B). UniProt has two entries for the short isoform because there are two potential methionine start codons, separated by two amino acids. A putative signal sequence is identified in the long TSLP isoform, with a predicted cleavage site after the threonine residue at amino acid 28, leaving a mature lfTSLP protein of 131 amino acids [2]. The calculated molecular weight (MW) of lfTSLP with, or without, the signal sequence is 18.1 kDa and 15 kDa, respectively, but in Western blotting the apparent MW is 23 kDa, probably due to post-transcriptional modifications (PTM) [5].

The N-terminal sequence of sfTSLP also contains a potential N-terminal signal sequence of 20 aa (SignalP, [8]). The MW of sfTSLP with or without signal sequence is 7.4 kDa (63 aa) or 7.1 kDa (60 aa) and 5.2 kDa (63 aa) or 4.8 kDa (60 aa), respectively. In Western blotting, the observed MW lies at 9 kDa, probably due to PTM [5]. The PTM might be glycosylation because two potential sites for N-linked glycosylation are present in the long isoform and one potential site is seen in the short isoform (Figure 1B). sfTSLP is predicted to consist of two α-helices (Figure 1C,D; [9]). As yet, onlya few studies have examined the expression of sfTSLP [5,6,10,11,12,13].

## 3. Expression and Regulation of TSLP Variants

**Figure 2:**
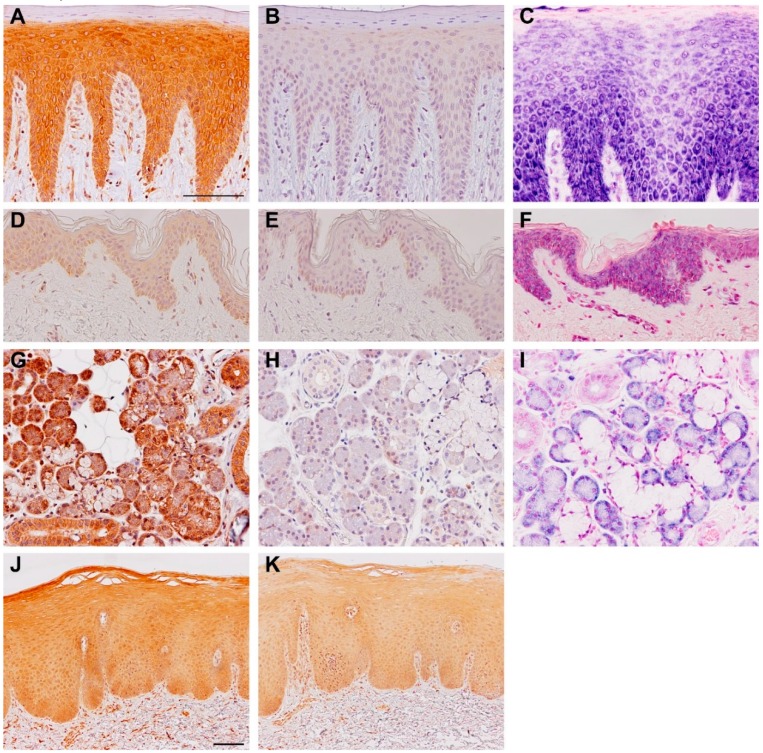
Immunohistochemical (IHC) staining and in situ hybridization (ISH) of sections of oral mucosa (**A**–**C**), skin (**D**–**F**), salivary gland (**G**–**I**), and smokeless tobacco (“snus”; **J**,**K**) for TSLP variants. Left column: IHC staining with anti-TSLP antibody recognizing both lfTSLP and sfTSLP (brown color). Middle column: IHC staining with anti-TSLP antibody recognizing lfTSLP only. As no specific staining is detected in (**B**,**E**,**H**), this means that the staining in (**A**,**D**,**G**) represents sfTSLP. In oral mucosa exposed to smokeless tobacco, lfTSLP is seen (**K**). Right column: ISH staining by use of sfTSLP-specific probe (blue color) which confirms strong expression of sfTSLP in oral mucosa and salivary gland, and weak expression in skin. Modified from [5].

Use of variant-specific reagents is necessary to study the expression of the two human TSLP variants separately. At the mRNA level, this differentiation can be obtained by the use of variant-specific primers, which are constructed based on unique mRNA sequences in the two transcript variants. Detection of variant-specific protein expression, however, requires an indirect approach. As there is a total overlap of sfTSLP with the lfTSLP amino acid C-terminal sequence, antibodies raised against sfTSLP epitopes will recognize both variants and the production of sfTSLP-specific antibodies is, therefore, not possible. On the other hand, it is possible to generate antibodies specific for lfTSLP, either by immunization with peptide sequences that lie within the specific lfTSLP sequence, or by retrieving monoclonals that recognize such sequences. Thus, distinguishing between sfTSLP and lfTSLP has to be overcome by comparing the combined content of lfTSLP and sfTSLP in samples, using one antibody that recognizes both forms, and another antibody that binds to the unique sequence of lfTSLP (a long-specific anti-TSLP antibody). This approach has been used both for Western blotting and immunohistology [5,6] (Figure 2).

The expression pattern of the two human isoforms is dependent on both tissue localization and disease state. Most of the TSLP literature of the last two decades has only focused on lfTSLP, as the translation of sfTSLP only recently has been documented [5]. The expression of TSLP has largely been associated with inflammatory conditions by which it was found to be upregulated. We now know that this was due to increased expression of lfTSLP [5,6]. In vivo, lfTSLP is upregulated in conditions such as atopic dermatitis, asthma, ulcerative colitis, and smokeless tobacco-exposed oral mucosa, while it is absent in healthy tissues (Figure 2) [5,6,10,14]. In vitro studies of cultured dermal and oral keratinocytes exposed to pro-inflammatory factors, such as interferon γ (IFN-γ), tumor necrosis factor α (TNF-α) in combination with interleukin 1β (IL-1-β), and polyriboinosinic:polyribocytidylic acid (poly(I:C)), show upregulation of lfTSLP mRNA and protein [5,15]. The intestinal epithelial cell line Caco-2, challenged with *Salmonella* *typhimurium*, shows upregulation of both mRNA and protein lfTSLP expression [6]. Finally, T_H_2 cytokines were found to be potent inducers of TSLP in human bronchial epithelial cells [16]. In normal nasal mucosa cultured in the presence of the inflammatory T_H_2 cytokines; IL-4, IL-13, and TNF-α, lfTSLP mRNA is upregulated [13]. Increased mRNA and protein TSLP expression were detected upon exposure of immunodeficiency virus in cervical epithelial cells [17] and exposure to poly(I:C) and a cocktail of IL-1 and TNF in airway epithelial cells [18]. Although not emphasized in these studies, the increased TSLP expression detected is presumably due to lfTSLP.

In contrast to lfTSLP, sfTSLP (mRNA and protein) is the predominant form of TSLP constitutively expressed in healthy tissues, including clinically healthy oral epithelium, skin epidermis, salivary glands, and gut epithelial cells (Figure 2) [5,6]. Under inflammatory conditions, sfTSLP appears to be downregulated as observed in lesional biopsy material from atopic dermatitis (AD) and in the intestine of patients with Crohn’s disease [6]. Exposure to *S.* *typhimurium* also downregulates sfTSLP mRNA and protein expression in Caco-2 cells [6]. To this date, sfTSLP protein expression has only been identified in the gut, skin, oral epithelium and salivary glands [5,6].

The divergent expression pattern for the two translated TSLP variants is consistent with the analysis of the human TSLP locus that reveals that the two variants are not alternatively spliced, but are derived from the activity of two separate, putative promotor regions [6]. The sfTSLP promotor appears to exhibit a high capacity to bind a number of different transcription factors, while the region upstream from the lfTSLP under steady-state conditions is relatively inert in most of the cell lines present in the UCSC database. Thus, under steady-state conditions, sfTSLP represents the homeostatic form of TSLP. In inflammation, lfTSLP is up- and sfTSLP is downregulated.

The expression and regulation pattern of TSLP in mice overlaps to a large extend that of human lfTSLP. A role of TSLP in human allergic diseases is well supported by a variety of mouse models [19,20,21,22] and increased lung tissue expression of TSLP has been detected in mice challenged with dsRNA [23]. In the steady state, TSLP expression in the skin of mice appears to be negatively regulated by retinoid X receptors (RXR) [24]. In the latter study, keratinocyte-specific ablation of RXRs resulted in upregulation of TSLP and development of AD-like skin inflammation. Further, the phenotype of mice lacking TSLP signaling (tslpr(−/−)) and challenged with human metapneumovirus (hMPV) show reduced lung infection and hMPV replication [25]. These mice displayed a decreased number of neutrophils, as well a reduction in levels of thymus and activation-regulated chemokine/CCL17, IL-5, IL-13, and TNF-α in the airways upon hMPV infection compared to WT mice.

## 4. Human TSLP Variants and Immunoregulation

### 4.1. Long-Form TSLP (lfTSLP)

**Figure 3:**
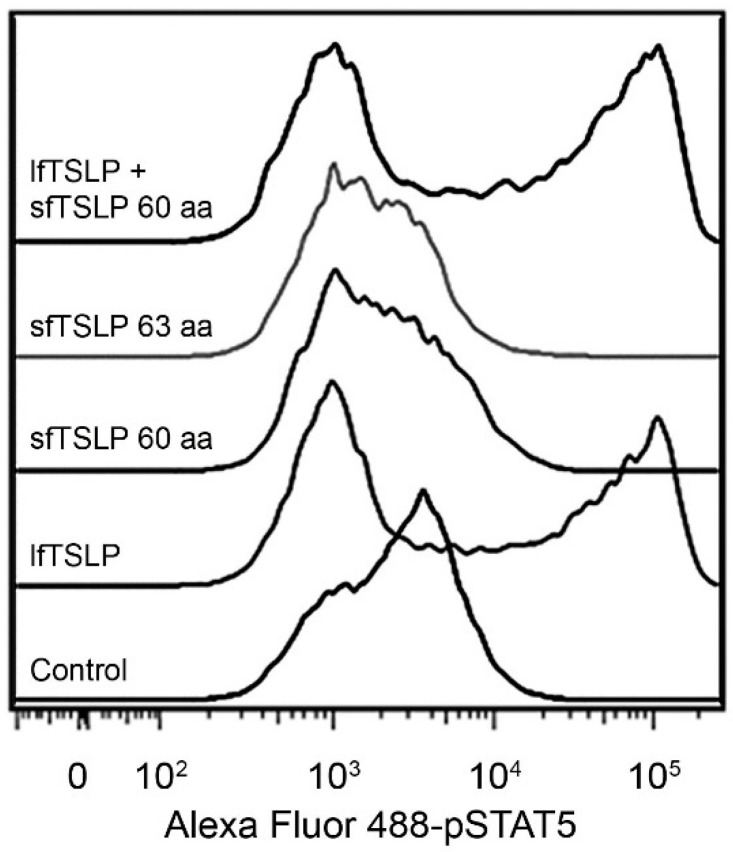
STAT5 phosphorylation in response to lfTSLP, 60 aa sfTSLP, 63 aa sfTSLP, or lfTSLP combined with sfTSLP in blood-derived CD1c myeloid DCs incubated with poly(I:C) for 24 h, and then treated with sfTSLP or/and lfTSLP for 15 min. Phosphorylation of STAT5 was assessed by flow cytometry. From [5].

lfTSLP is closely related to IL-7, with which it shares an overlapping, but not identical, biological profile, and binds to a heterodimeric receptor complex consisting of the IL-7 receptor α-chain (IL-7Rα) and the TSLP receptor chain (TSLPR) [2,26]. The functional receptor for lfTSLP is expressed on both hematopoietic and non-hematopoietic cell lineages including DCs, T cells, B cells, natural killer cells, monocytes, basophils, eosinophils, and epithelial cells [3,18,19,27,28,29,30,31,32]. Activation of the TSLP receptor has been shown to signal through multiple signal transducer and activator of transcription (STAT) proteins, including STAT 1, 3, 4, 5, 6, and Janus kinase (JAK) 1 and 2 in peripheral blood-derived CD11c^+^ DCs (Figure 3) [5,33,34].

lfTSLP has an impact on several immune functions and has, as mentioned above, been associated with immune disorders, such as allergic diseases and intestinal inflammation. Co-culture of lfTSLP-stimulated DCs with allogeneic CD4^+^ T cells results in the generation of inflammatory Th2 cells producing classical Th2 cytokines including IL-4, IL-5, IL-13, but in contrast to conventional Th2 cells, these cells also produce TNF-α and not IL-10 [14]. This inflammatory Th2 phenotype is induced through the upregulation of OX-40 ligand expression on lfTSLP-treated DCs [14,35]. Accordingly, in atopic dermatitis (AD), lfTSLP protein is not detectable in non-lesional skin in AD patients, while it is highly expressed in acute and chronic AD lesions [14]. In allergic rhinitis, TSLP treatment of CD1c^+^ DCs potently augments allergen-specific T_H_2 memory responses [13].

In contrast to its role in inflammation, TSLP has also been suggested to have homeostatic, tolerogenic functions [36,37]. It was, however, at that time unknown that the sfTSLP peptide is also translated, and that this peptide has an inhibiting effect on DCs [5]. After a re-evaluation of earlier results and further investigations, it is now clear that sfTSLP is responsible for this effect in the intestine [6].

### 4.2. Short-Form TSLP (sfTSLP)

sfTSLP is constitutively expressed by several types of epithelial cells, as described above. sfTSLP appears to act on DCs on which it inhibits cytokine secretion [6]. sfTSLP does not bind to the TSLPR because it is not capable to block binding of lfTSLP to this receptor (Figure 3) [5,6]. The specific receptor for sfTSLP is currently unknown. sfTSLP induces phosphorylation of p38α, extracellular signal-regulated kinase 1/2, and Lyn, but has no effect on STAT5 phosphorylation (Figure 3) [5,6]. Very little else is yet known about the immunoregulatory action of sfTSLP. As sfTSLP can be downregulated by inflammation, this might contribute to an aggravation of local infection in view of its antimicrobial activity (see below).

## 5. Human TSLP Variants as Antimicrobial Peptides

AMPs can be classified in a variety of approaches [38], fitting into one of four major structural classes: (1) linear peptides that may adopt α-helical conformation upon bacterial binding; (2) β-sheet peptides; (3) extended peptides with over-representation of specific amino acid residues; or (4) looped peptides [39,40,41]. However, dermcidin, an AMP secreted by sweat glands [42], is often classified based on its anionicity.

**Figure 4:**
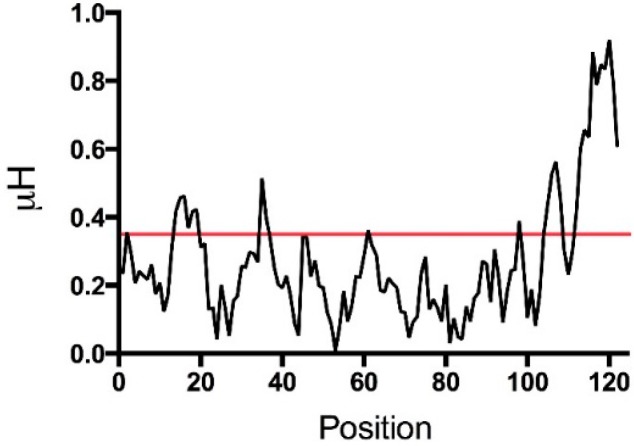
Plot of hydrophobic moment (μH) for the mature lfTSLP (131 amino acids).

A common characteristic of AMPs is the propensity to form helical structure [40,43]. It has been previously reported that TSLP contains several predicted helical regions [3] (Figure 1C). Thus, both sfTSLP and lfTSLP are cationic peptides with regions that could display α-helical conformation. Moreover, analysis of the mature lfTSLP (131 amino acids) reveals that it may harbor qualities required for antimicrobial activity at physiological conditions, such a positive net charge of +11 and a theoretical isoelectric point (pI) of 9.63 (calculated by using the Protparam tool; Swiss Institute of Bioinformatics, Lausanne, Switzerland). Furthermore, analysis of the hydrophobic moment (μH) revealed a region, likely in the C-terminal part of TSLP, with conspicuous amphiphathic properties (Figure 4) (calculated by using the European Molecular Biology Open Software Suite (EMBOSS); The Sanger Centre, Wellcome Trust Genome Campus, Hinxton, Cambridge, UK).

**Figure 5:**
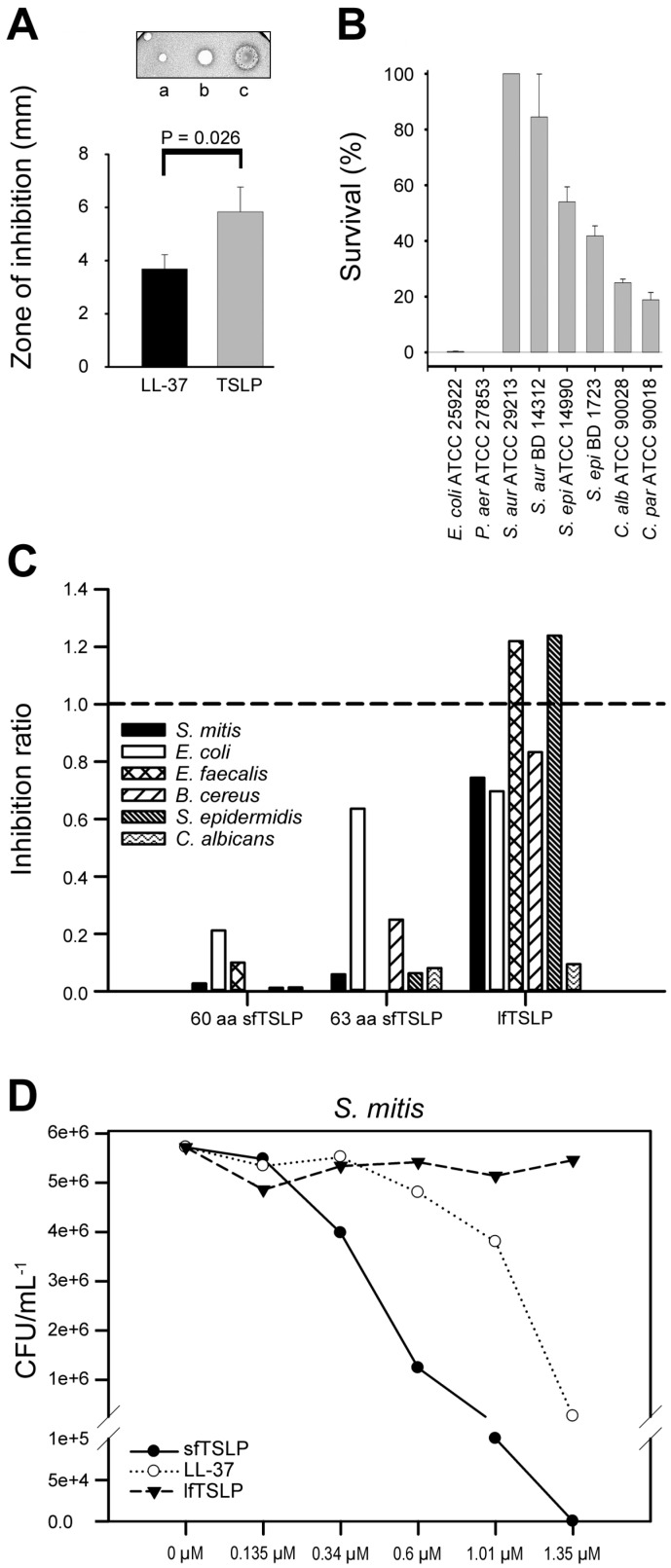
Antimicrobial activity of short and long forms of thymic stromal lymphopoietin (sfTSLP and lfTSLP). (**A**) lfTSLP exhibited a larger zone of inhibition of growth of *Escherichia coli* ATCC 25922 in comparison with LL-37: (a) control; (b) 10 µM LL-37; and (c) 10µM TSLP. Mean values and standard deviations (n = 4). (**B**) In a viable count assay, indicated bacterial (*Escherichia coli, Pseudomonas aeruginosa, Staphylococcus aureus*, and *Staphylococcus epidermidis*) and fungal isolates (*Candida albicans* and *Candida parapsilosis*) were subjected to 2 µM of TSLP. The number of cfu was registered. (**C**) Suspensions of the indicated bacterial and fungal species were treated for 2 h with 60 amino acid (aa) sfTSLP, and 63 aa sfTSLP or lfTSLP peptide at a concentration of 1.35 mM before being plated on agar. Colony-forming units per ml were determined after incubation overnight. The values were normalized to the levels obtained without the addition of test peptides (broken line). (**D**) Suspensions of *Streptococcus mitis* were treated with equimolar concentrations of 60 aa sfTSLP, LL-37, or lfTSLP and analyzed as in C. From: [7] (**A**,**B**) and [5] (**C**,**D**).

**Figure 6:**
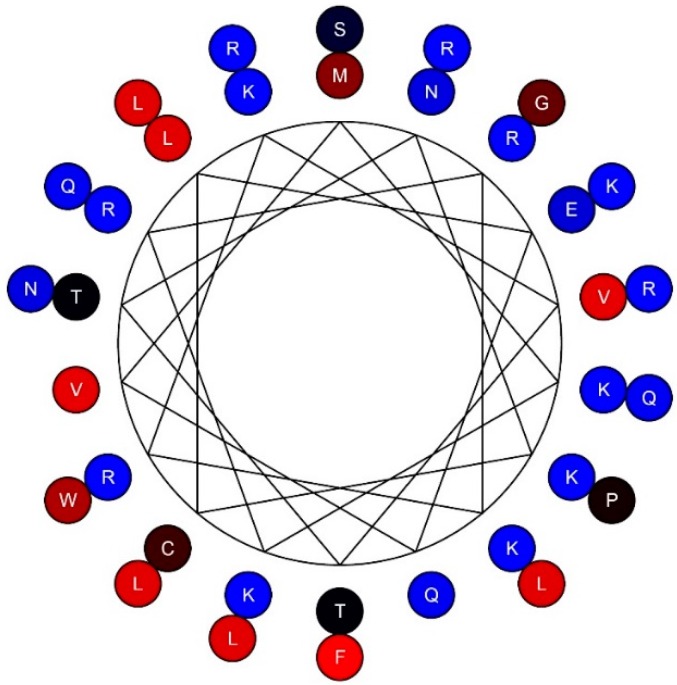
Helical structure of MKK34. A helical wheel projection was constructed using the amino acid sequence of MKK34.

Our data displayed antimicrobial activity of TSLP against both bacteria and fungi [5,7] (Figure 5A,B). To further investigate the antimicrobial properties of TSLP and which regions of the molecule exhibited the antimicrobial effects, overlapping 20-mer peptides were synthesized [7]. The experiments showed that the antimicrobial effect preferentially was located in regions of the C-terminal part of TSLP [7]. When a 34 aa long synthetic peptide (MKK34; Figure 1B) spanning the C-terminal part of TSLP was tested for antimicrobial activities, it exerted potent antimicrobial activity, both in the presence of human plasma and in physiological salt conditions [7]. MKK34 contains predicted regions that could display α-helical conformation. A helical wheel projection of MKK34 is visualized in Figure 6.

The findings that the main antimicrobial activity of TSLP is located in its C-terminal part is particularly relevant since both MKK34 and sfTSLP are found in this region, and sfTSLP is translated and constitutivelyexpressed in normal tissues [5]. In our studies, both forms of TSLP and MKK34 were found to have antimicrobial action against Gram-positive and Gram-negative bacteria, and fungi, stronger than the well-characterized AMP LL-37 [5,7] (Figure 5B,C). sfTSLP exerted potent antimicrobial activity against all the tested species, including *Streptococcus mitis, Escherichia coli, Enterococcus faecalis, Bacillus cereus, Staphylococcus epidermidis*, and *Candida albicans* [5] (Figure 5C). Moreover, addition of polyclonal anti-TSLP antibody to sfTSLP before it was incubated with *S. mitis*, reduced the antimicrobial activity by about half, showing that the reduction in colony-forming units per mL was specifically due to the action of sfTSLP [5]. Dose-response curves using *S. mitis* showed that the effect of sfTSLP was stronger than that of LL-37 (Figure 5D). Furthermore, the susceptibility of isolates of *Staphylococcus aureus*, *S. epidermidis*, *E. coli*, and *Pseudomonas aeruginosa* to MKK34 was tested in antimicrobial assays [7]. The Gram-positive isolates were generally less susceptible to MKK34 in comparison to Gram-negative bacteria [7].

AMPs are reported to possess different antibacterial spectrums. The well-characterized AMP LL-37 has a broad spectrum whereas psoriasin preferentially kills *E. coli* [44,45]. Considering that TSLP is released in response to microbial stimulation of epithelial cells [18], our findings suggest that TSLP and TSLP-derived peptides, such as MKK34 and sfSTLP, exert broad antimicrobial activity on Gram-negative bacteria, Gram-positive bacteria, as well as fungi, that are of importance in host defense [5,7]. Moreover, MKK34 may be a contributor to the in vivo resistance of human skin to Gram-negative bacterial colonization and infection and hypothetically support the maintenance of preferentially Gram-positive bacterial (*S. epidermidis*) colonization at the human skin.

**Figure 7:**
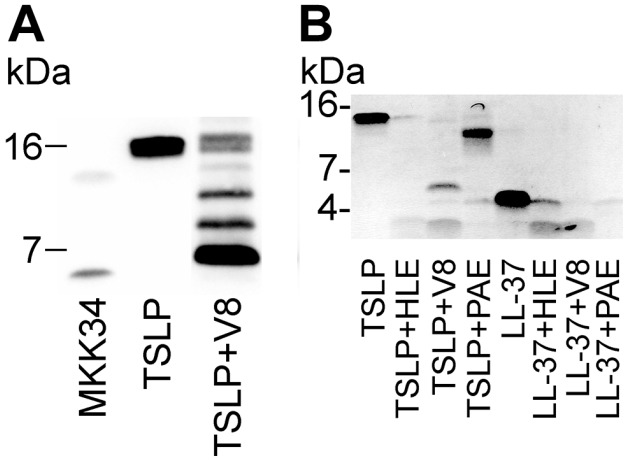
Enzymatic digestion of TSLP. (**A**) TSLP was digested with S. aureus V8 proteinase, and cleavage products were visualized by Western blot analysis using polyclonal antibodies against human TSLP. Products produced by V8 cleavage of TSLP revealed a major immunoreactive protein fragment at about 16 kDa. (**B**) TSLP and LL-37 were incubated with and without human neutrophil (leukocyte) elastase (HLE), *S. aureus* V8 proteinase or *Pseudomonas aeruginosa* elastase (PAE) and analyzed under non-reducing conditions by SDS-PAGE. From ref. [7].

Several classical and recently discovered AMPs, such as LL-37 and AMPs derived from larger proteins, are generated by proteolytic processing resulting in bioactive fragments that exert antimicrobial effects [46,47,48,49]. As mentioned before, TSLP is highly expressed by keratinocytes in atopic eczema. Moreover, AD skin is frequently colonized by *S. aureus* and characterized by a chronic inflammatory infiltrate [14,50]. Therefore, it is tempting to speculate that AD skin cleavage of TSLP by proteases (both endogenous and bacterial) produces small antimicrobial fragments. To test this, we incubated TSLP in the presence of neutrophil (leukocyte) elastase (HLE), which is produced by leukocytes during inflammation, as well as in the presence of different bacterial derived proteases. When analyzed by SDS-PAGE, the incubation products revealed degradation of TSLP by both HLE and the bacterial proteases (*P. aeruginosa* elastase, *S. aureus* V8) (Figure 7). The *S. aureus* V8 proteinase degraded TSLP into three distinct fragments, the major peptide fragment (fragment I) being derived from the C-terminal part and comprising 42 amino acids (Thr88-Lys129), encompassing the previously characterized synthetic MKK34 peptide. When the V8 degradation products were tested, the results showed similar antibacterial activity of the degradation product against the Gram-negative bacteria *E. coli* as the holoprotein TSLP [7].

**Figure 8:**
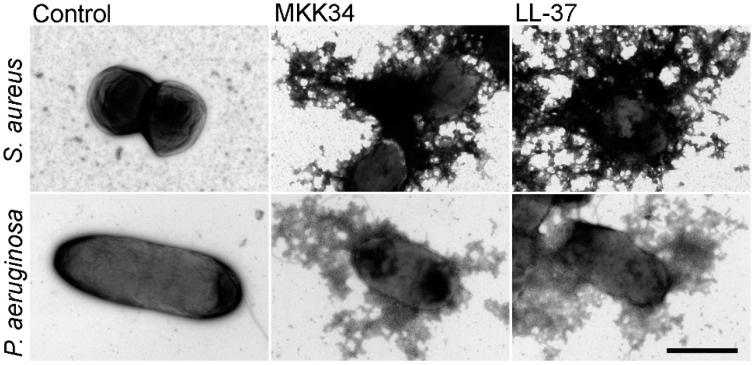
Electron microscopy analysis. *Staphylococcus aureus* and *Pseudomonas aeruginosa* were incubated with 30 μM of MKK34 and LL-37 for 2 h at 37 °C and visualized by negative staining. Scale bar 1 μm. Control: buffer control. From [7], image courtesy of Matthias Mörgelin, Lund University, Lund, Sweden.

AMPs are known to exert their effects by different mechanisms: some are membrane-active and others are not [43]. To investigate if MKK34 exerted membrane active properties, we performed liposome leakage models and fluorescence studies on peptide-treated bacteria. This showed that MKK34 exerted membrane-penetrating effects on bacterial membranes of *E. coli*, as well as on liposomes [7]. Moreover, electron microscopy analysis revealed severe membrane damage of MKK34-treated bacteria (Figure 8).

The redox state of AMPs can be a determining factor for their activity: reduction of disulphide-bridges in human beta-defensin-1 (hBD-1) vastly potentiates its antimicrobial effect and free cysteines in the carboxy terminus seem important for the bactericidal effect [51]. Of the three disulphide bridges in lfTSLP (Cys34-Cys110, Cys69-Cys75, and Cys90-Cys137; [2]), one is present in sfTSLP (Cys90-Cys137). It will be interesting to examine whether the redox state of cysteine residues in sfTSLP can affect its structure and antimicrobial properties. It also remains to be confirmed that sfTSLP occurs in sufficiently high concentrations in mucosal secretions and exfoliated skin to be effective as an antimicrobial agent.

Whether murine TSLP exhibits antimicrobial properties has not yet been investigated.

## 6. Conclusions

Taken together, the latest studies on TSLP have shown (1) that human TSLP is translated in two forms; (2) that the short form is constitutively expressed in a steady-state, while the long form is absent; (3) that the long form is induced by inflammation, while the short form appears to be downregulated by inflammation; (4) that both forms exhibit potent AMP activity, with sfTSLP exhibiting the strongest activity; (5) that the AMP activity is primarily localized in the C-terminal part of both forms; and (6) that the C-terminal part of TSLP a has penetrating effect on bacterial membranes.

As human sfTSLP is constitutively expressed at major barrier surfaces consisting of skin and mucosa, it is expected to play an important role against infection and regulation of inflammation at these sites. lfTSLP has a broader role in the defense against infection because it participates in the regulation of various immune activities, but it also preserves its antimicrobial functions.
